# Evaluating Intervention Fidelity: An Example from a High-Intensity Interval Training Study

**DOI:** 10.1371/journal.pone.0125166

**Published:** 2015-04-22

**Authors:** Kathryn L. Taylor, Matthew Weston, Alan M. Batterham

**Affiliations:** 1 Health and Social Care Institute, Teesside University, Middlesbrough, United Kingdom; 2 Department of Sport & Exercise Sciences, School of Social Sciences, Business & Law, Teesside University, Middlesbrough, United Kingdom; University of Rome, ITALY

## Abstract

**Aim:**

Intervention fidelity refers to the degree to which an experimental manipulation has been implemented as intended, but simple, robust methods for quantifying fidelity have not been well documented. Therefore, we aim to illustrate a rigorous quantitative evaluation of intervention fidelity, using data collected during a high-intensity interval training intervention.

**Design:**

Single-group measurement study.

**Methods:**

Seventeen adolescents (mean age ± standard deviation [SD] 14.0 ± 0.3 years) attended a 10-week high-intensity interval training intervention, comprising two exercise sessions per week. Sessions consisted of 4-7 45-s maximal effort repetitions, interspersed with 90-s rest. We collected heart rate data at 5-s intervals and recorded the peak heart rate for each repetition. The high-intensity exercise criterion was ≥90% of individual maximal heart rate. For each participant, we calculated the proportion of total exercise repetitions exceeding this threshold. A linear mixed model was applied to properly separate the variability in peak heart rate between- and within-subjects. Results are presented both as intention to treat (including missed sessions) and per protocol (only participants with 100% attendance; n=8).

**Results:**

For intention to treat, the median (interquartile range) proportion of repetitions meeting the high-intensity criterion was 58% (42% to 68%). The mean peak heart rate was 85% of maximal, with a between-subject SD of 7.8 (95% confidence interval 5.4 to 11.3) percentage points and a within-subject SD of 15.1 (14.6 to 15.6) percentage points. For the per protocol analysis, the median proportion of high-intensity repetitions was 68% (47% to 86%). The mean peak heart rate was 91% of maximal, with between- and within-subject SDs of 3.1 (-1.3 to 4.6) and 3.4 (3.2 to 3.6) percentage points, respectively.

**Conclusions:**

Synthesising information on exercise session attendance and compliance (exercise intensity) quantifies the intervention dose and informs evaluations of treatment fidelity.

## Introduction

Intervention fidelity—the extent to which an experimental manipulation has been implemented as intended, in a comparable manner to all participants [1], is integral to the internal validity of intervention-based trials [2,3]. An evaluation of fidelity helps prevent incorrect conclusions being drawn about the effect (positive or negative) of an intervention on a given outcome [4]. However, despite many journals endorsing trial reporting guidelines such as the CONSORT (Consolidated Standards of Reporting Trials) statement [5], the quality of intervention reporting remains unacceptably poor [6]. This issue is especially problematic in exercise trials, as intervention findings are of very little value without precise, thorough and in-depth information about the exercise training itself [7].

When reporting exercise interventions authors frequently provide data on training session attendance, usually as a percentage of the total number of prescribed sessions, yet regularly fail to indicate the extent to which participants complied with the prescribed exercise intensities [8]. Intensity is very often the essential component of an exercise intervention; therefore intensity monitoring would provide an objective measure of the extent to which participants complied with the prescribed exercise dose. Heart rate monitoring provides a simple and reliable measure of exercise intensity in both laboratory and field settings [9] and is regarded as one of the best and most popular ways to monitor exercise intensity [10]. It is therefore surprising that so few exercise training studies present heart rate data to demonstrate the extent to which participants complied with prescribed intensities. Of the studies that have presented intervention heart rates [8,11] only overall mean and between-subject standard deviation (SD) data are reported. These values do not, however, provide a clear indication of the fidelity of the intervention. Furthermore, repeated exercise sessions performed across an intervention period will give rise to between-subject and within-subject variability in the exercise intensity response. As the observed between-subject SD provides no robust information pertaining to the within-subject variability, and overestimates the true between-subject variability, the reader is left unable to establish whether the content and process of the intervention was consistent throughout the trial [1]. The appropriate use of linear mixed modelling would overcome this shortcoming, by properly separating the between- and within-subject variability and quantifying the degree of consistency in exercise intensity across the intervention time period.

Evaluations of fidelity in exercise training interventions should address session attendance and compliance (meeting the prescribed exercise intensity), as this interaction constitutes the ‘dose’ of the intervention and influences the physiological response to chronic exercise training. Fidelity may be examined within both intention to treat and per protocol frameworks [12]. In a randomised controlled trial, an intention to treat approach involves analysis of all participants in their original assigned groups (intervention and control), regardless of whether participants have actually complied with the trial protocol. A per protocol analysis, on the other hand, incorporates only the data of those participants who followed the study protocol strictly. Consequently, to quantify the overall dose of the intervention, intention to treat fidelity analysis should include all participants irrespective of their attendance and compliance. In contrast, a per protocol fidelity analysis should involve only those participants who attended all of the prescribed sessions. Both approaches are informative in a full exploration of fidelity.

The purpose of this study therefore was to illustrate a rigorous evaluation of intervention fidelity, using both intention to treat and per protocol frameworks. To illustrate our quantitative approach we have utilised data collected during a 10-week school-based high-intensity interval training intervention for our analysis. Therefore, while we have reported a full description of the high-intensity interval training intervention, our evaluation is confined to the fidelity of the intervention, not the intervention effects per se.

## Methods

Project FFAB (Fun Fast Activity Blasts) was an exploratory controlled before-and-after study on the effect of a 10-week school-based high-intensity interval training intervention on cardio-metabolic risk markers and physical activity levels in adolescents. High-intensity interval training is characterised by brief, intermittent bouts of intense exercise, alternated with periods of rest or low-intensity active recovery [13]. Over the last decade, there has been a resurgence of scientific interest in the efficacy of high-intensity interval training as a time-efficient way of improving health and fitness outcomes [13,14]. Project FFAB took place in four secondary schools in the Tees Valley region of North East England. Two schools were assigned to the high-intensity interval training intervention (n = 41) and two acted as controls (n = 61). Participants at one of the two intervention schools were required to wear heart rate monitors at every high-intensity interval training session; therefore, we modelled the heart rate data recorded for these participants (n = 17; 7 females) to illustrate our fidelity assessment method. Ethics clearance for the study was granted by Teesside University Research Ethics and Governance Committee. Parents and school pupils gave written informed consent and assent, respectively, for participation in the study.

**Fig 1:**
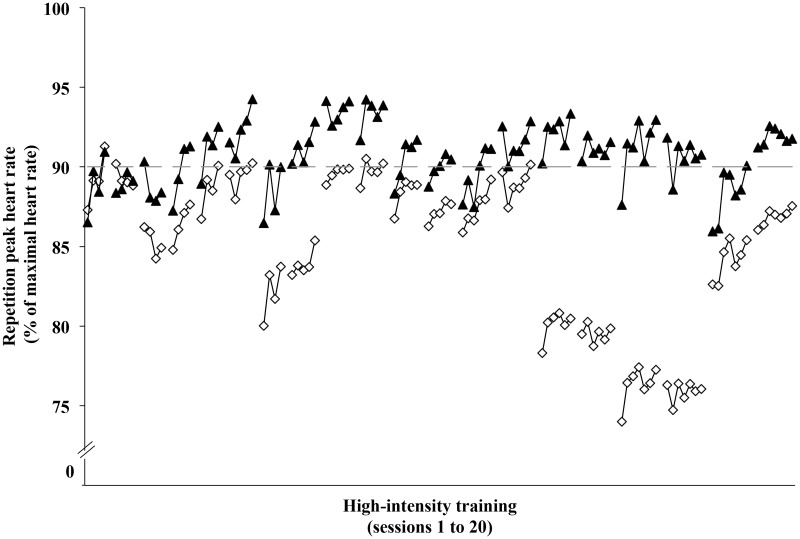
Repetition mean peak heart rates during the high-intensity interval training sessions. ▲ **represent the PP data; ◇ represent the ITT data; ── indicates our threshold for high-intensity [90%HRmax]. Error bars have been omitted for figure clarity.**

The high-intensity interval training sessions were led by the first author. Participants (mean age ± SD 14.0 ± 0.3 years) performed two sessions per week in physical education lessons undertaken during the school term. Sessions commenced with a 5-min warm-up and culminated with a 5-min whole body cool down. Following the warm-up, participants performed four repetitions of 45-s of maximal effort exercise (boxing, dance, soccer and basketball drills) with 90-s rest in between each. The intervention activities were chosen based on data collected in pre-intervention focus groups with adolescent school students. These were conducted to aid the intervention design and maximise the likelihood of participants attending and complying with the intervention. Here, participants expressed a desire for the intervention to incorporate a variety of activities (basketball, boxing, dance and soccer) with the exercise mode rotated on a session-by-session basis [15]. Data collected during a pilot of Project FFAB confirmed that drills based on these activities were capable of eliciting a high-intensity dose (e.g. peak heart rate ≥90% of maximal) [15]. At the trial onset, it was intended that the number of repetitions performed across the two sessions would increase by two every two weeks, thus ensuring progression across the intervention. During the first two weeks, however, it was apparent that the participants were struggling with the workload. In an attempt to alleviate this, the number of repetitions increased by only one after weeks 2 and 4. From week 6 onwards, the number of repetitions performed across the two sessions increased by two every two weeks. The total number of repetitions delivered across the 10-week intervention was 106 (see Fig 1).

During each exercise repetition, participants were verbally motivated to provide “all-out/maximal efforts”. We checked heart rate during and after each session to encourage intensity compliance. Exercise time across the high-intensity interval training sessions was 279.5 min, and the total time spent performing the 45-s repetitions was 79.5 min. At the start of each session, participants were fitted with a heart rate monitor (Polar RS400, Polar Electro, Finland). We collected heart rate data at 5-s intervals throughout the exercise sessions. Determination of compliance is difficult [16]; therefore, a cut-point of ≥90% of maximal heart rate was used as our criterion for satisfactory compliance to high-intensity exercise, reflecting that used in previous work on high-intensity interval training [17].

Following each training session, we downloaded each heart rate file into the Polar ProTrainer software (Polar Electro, Kempele, Finland). Initially, all files were visually inspected and values outside the normal physiological range expected for the participants (>220 beats·min^-1^or <40 beats·min^-1^) were corrected using the software’s error correction function. We then derived the peak heart rate of each 45-s exercise repetition from each individual file. These values were expressed as a percentage of the individual’s maximal heart rate. Participants’ maximal heart rates were determined as the highest 5-s value recorded during the high-intensity interval training sessions, or during the multistage fitness test completed for the baseline data collection [18]. This method can enable a more accurate determination of maximal heart rate than a single maximal fitness test [11].

For the intention to treat analysis, a value of 40% of maximal heart rate was imputed for cases where heart rate data were missing due to participant absence. We elected to use this value as, in this study population, 40% of maximal heart rate represents no additional physiological loading (e.g. ~80 beats·min^-1^). It was also necessary to account for the few instances where heart rate data were missing due to equipment malfunction. Although more sophisticated approaches have been proposed [19], in the case of missing heart rate data for one or more repetitions we imputed the mean of the participant’s peak heart rates in the prior repetitions (or subsequent repetitions in the case of missing data on the first repetition in a session). For the intention to treat analysis there were a total of 1802 high-intensity exercise repetitions (106 possible repetitions for each of the 17 participants). For the per protocol analysis, there were a total of 848 repetitions (106 repetitions for each of the 8 participants attending all 20 prescribed sessions).

Heart rate data for each 45-s high-intensity exercise repetition were analysed using SPSS Statistics software (v.21, Armonk, NY: IBM Corp). For both the intention to treat and the per protocol approaches, the following analyses were conducted. For each individual participant, we first determined the proportion of repetitions in which the high-intensity exercise criterion was attained. We then derived the median and interquartile range of these individual proportions. To provide the correct overall between- and within-subject variability (expressed as an SD) in peak heart rate across the repeat 45-s repetitions, we applied a linear mixed model with sex, session, and repetitions (nested within a session) included as fixed effects. Peak heart rate (percentage of maximal) was treated as a continuous variable, with model specification checked using residuals plots. The residuals from the linear mixed models were well behaved, indicating that treating bounded percentage heart rate data as continuous was acceptable. Data are expressed as mean ± SD, with uncertainty in the estimates expressed as 95% confidence intervals.

## Results

One participant dropped out of the trial after week 6, having attended every session up to that point (n = 12). Eight participants completed all 20 sessions. Three completed 18 sessions, four completed 16 and one completed 14. Reasons for participant absence from sessions were illness and/or individual holidays.

For the intention to treat analysis, the median (interquartile range) of the proportions of repetitions for individual participants wherein the high-intensity exercise criterion was attained was 58% (42% to 68%). The mean for peak heart rate across all repetitions was 85% of maximal. The mixed model analysis revealed that the between- and within-subject SDs were 7.8 (95% confidence interval 5.4 to 11.3) percentage points and 15.1 (14.6 to 15.6) percentage points, respectively (Fig 1). For the per protocol analysis, the median (interquartile range) of the proportions of repetitions in which individual participants attained our high-intensity exercise criterion was 68% (47% to 86%). The mean peak heart rate was 91% of maximal, and the between- and within-subject SDs were 3.1 (-1.3 to 4.6) percentage points and 3.4 (3.2 to 3.6) percentage points, respectively (Fig 1).

## Discussion

Across health and sport performance interventions, fidelity measures should be employed to ensure that trials are fully evaluated and interventions are not accepted or rejected on the basis of inadequate or incomplete information [20]. Using heart rate data collected during a 10-week school-based high-intensity interval training trial, our aim was to provide a framework for the rigorous evaluation of intervention fidelity using intention to treat and per protocol analyses.

We place inferential emphasis on the intention to treat analysis, as this is the least-biased and preferred approach to subsequent analysis of randomised trial data. This analysis indicated that the overall fidelity of the intervention was moderate at best, with the high-intensity heart rate criterion attained in a little over half of the 106 possible repetitions per participant. The between-subject variability was around 9% of the mean and within-subject variability (from repetition to repetition) approximately 18% of the mean, revealing that the stimulus was not applied consistently across the whole intervention. This moderate fidelity was due in large part to missed sessions, rather than poor compliance in attended sessions. Indeed, of a possible total of 340 participant-sessions (17 participants × 20 sessions) 36 were missed (c. 11%). Given that our quantitative evaluation of intervention fidelity represents the first of its kind, reconciliation with the work of others is not yet possible. Were future studies to incorporate our analytical approach, the development and implementation of robust qualitative inferences for the assessment of intervention fidelity would be possible.

The per protocol analysis revealed that, on average, a high-intensity exercise stimulus (~90% of maximal heart rate) was complied with by those participants attending all exercise sessions. Moreover, the criterion for high-intensity exercise was attained for around two-thirds of the intervention repetitions. The high-intensity stimulus was applied consistently across the sample (demonstrated by the small between-subject SD of ~3% of the mean), throughout the intervention (indicated by the small within-subject SD of ~4% of the mean). Taken together, these findings demonstrate the high quality of the delivery of the high-intensity interval training intervention, and its receipt and enactment by the participants.

The contrasting findings of the intention to treat and per protocol analysis are consistent with the observation that estimated effects of an intervention are often lower when derived from an intention to treat analysis, due to the inclusion of participants that were non-adherent to, or deviated from the protocol [12]. In the context of this study, the former refers to participants that missed sessions, and the latter to those that attended but did not perform at the required intensity. Clearly our intention to treat analysis was influenced by the inclusion of a heart rate value (40% of maximal) representing non-attendance. Both intention to treat and per protocol approaches do, however, provide valuable information pertaining to the evaluation of intervention fidelity. Indeed, per protocol analysis provides an estimate of the maximal exercise dose attainable from the intervention given full attendance, whereas intention to treat analysis reflects the success of real-world implementation.

An additional potential use of fidelity data is in the intention to treat analysis of outcome data in a randomised controlled trial. For example, using linear mixed modelling, the proportion of total possible repetitions (106 in the current case) ≥90% of maximal heart rate could be included as a covariate in a secondary analysis to determine the extent to which fidelity explained variability in individual responses to the intervention. Using the observed relationship between fidelity and outcome, the effect of the intervention under ideal conditions of full attendance and compliance could be predicted, providing valuable information for a post-study process evaluation.

We must acknowledge two main limitations to our study. First, the sample size is small. However, our intention was to use the data gathered from just one intervention site to illustrate simply our methodological approach to fidelity assessment. Secondly, participants’ heart rates were used to confirm the high-intensity nature of the exercise sessions. It should be noted that the degree of compliance to an intervention cannot be established perfectly [16]. Indeed, it has been suggested that the usefulness of heart rate monitoring for controlling and adjusting the intensity of a high-intensity interval training session, rather than a prolonged submaximal one, might be limited [21]. This is largely due to the well-known heart rate lag at exercise onset which, compared to the oxygen uptake response, is much slower [22]. As such, while heart rate is expected to reach maximal values for exercise at or below the speed/power associated with maximal oxygen uptake, this is not always apparent, particularly for very short (<30 s) [23] and medium-long intervals [24]. This phenomenon might explain why a recent field based high-intensity interval training study did not measure participants’ heart rate during repeated 30-s high-intensity intervals [25]. In our trial, it may be that the 5-min warm-up was of insufficient duration and intensity, or the interim period between warm-up cessation and exercise too long, given that for the per protocol analysis the initial exercise repetition was the lowest for 15 of the 20 high-intensity interval training sessions. Notwithstanding these suppositions, we have shown that our participants’ heart rates peaked at ~90% of individual maximum within a 45-s repetition, despite the acknowledged heart rate lag. Our intensity estimates may therefore be conservative. This assertion is supported by the fact that mean heart rate responses were averaged across the 45-s repetitions, and therefore did not include any of the recovery period. This therefore avoided an overestimation of physiological load, which can occur when heart rate continues to rise after exercise cessation [24].

We have provided a framework for evaluating fidelity in the context of a high-intensity interval training trial for adolescents. Our model could, however, be used across disciplines for any outcome with a continuous variable (e.g. number of steps, power output, distance, velocity, ratings of perceived exertion) where the researcher wishes to evaluate the between- and within-subject variability and gauge the proportion of participants meeting their compliance criterion. Performing both intention to treat and per protocol analyses permits a richer understanding of the degree of intervention fidelity and the quality of intervention delivery, receipt and enactment. This rigorous approach therefore provides greater insight into the true relationship between exposure (the intervention) and outcome. Furthermore, it has been emphasised that fidelity might be under greater threat during complex interventions, where, for example multiple repeat sessions are delivered [3] and the location of the intervention varies [26]. Given that both these scenarios are commonplace across health and sport performance intervention studies, frameworks aiding the assessment of fidelity are timely.

## Conclusions

Our study has provided the first example of how intervention fidelity can be evaluated objectively as a composite measure of both attendance and compliance. Fidelity was examined in the context of a school-based high-intensity interval training intervention, and has highlighted the need to address intervention fidelity using intention to treat and per protocol analysis frameworks. We hope our model can serve as a framework for evidencing intervention fidelity in clinical, field- and laboratory-based sport and exercise medicine research.

## Supporting Information

S1 Dataset Study Data .
(XLSX)
